# Epidémiologie de la lèpre au Tchad de 2015 à 2019

**DOI:** 10.11604/pamj.2022.41.120.32283

**Published:** 2022-02-10

**Authors:** Abakar Kirga Kabo, Kaiwa Kaman, Djamalladine Mahamat Doungous, Lamine Ouedraogo, Mahamat Abakar, Sylvain Godreuil, Véronique Penlap Beng

**Affiliations:** 1 Ecole des Sciences de la Santé, Université Catholique d’Afrique Centrale, Yaoundé, Cameroun,; 2 Laboratoire de Bactériologie, Centre Hospitalier Universitaire de Montpellier, Montpellier, France,; 3 UMR-MIVEGEC (IRD 224, CNRS 5290, Université de Montpellier), Montpellier, France,; 4 Programme National de Lutte Contre la Lèpre, N´Djamena, Tchad,; 5 Département des Sciences Biomédicales et Pharmaceutiques, Institut National Supérieur des Sciences et Techniques d´Abéché, Abéché, Tchad,; 6 Université de Yaoundé I, Yaoundé, Cameroun

**Keywords:** Lèpre, épidémiologie, *Mycobacterium leprae*, Tchad, Leprosy, epidemiology, Mycobacterium leprae, Chad

## Abstract

**Introduction:**

la lèpre est une maladie présente au Tchad, inégalement répartie. Depuis 1997, la prévalence annuelle nationale est inférieure à 110000 habitants, seuil d´élimination fixé par l´Organisation Mondiale de la Santé (OMS). Le but de cette étude est de décrire les tendances épidémiologiques de la lèpre au Tchad entre 2015 et 2019, afin de fournir les données nécessaires pour l´élaboration de stratégies de lutte plus efficace contre la lèpre.

**Méthodes:**

il s´agissait d´une étude rétrospective descriptive sur les cas de lèpre survenus entre 2015 et 2019 à l´échelle nationale à partir de la base de données du Programme National de Lutte contre la Lèpre au Tchad (PNLLT).

**Résultats:**

au total, 1896 nouveaux cas de lèpre ont été détectés au Tchad entre 2015 et 2019, la proportion des patients âgés de 1 > 5 à 70 ans et celle des enfants de moins de 15 ans étaient de 92.08% et 7.92%. Le sexe ratio (H/F) était de 1.68. Le taux de détection moyenne annuelle est de 2,6/100 000, un taux moyen de 83.10% de cas de lèpre multibacillaire, et 20.38% d´incapacité de degré 2 (ID2) parmi lequel 0.92% ID2 chez les enfants de moins de 15 ans en moyenne. Cependant, notre étude a relevé cinq districts surendémiques (Adré, Abéché, Aboudeia, Koukou, et Bebedjia) en 2019 où le taux de prévalence est supérieur à 1/10000 habitants.

**Conclusion:**

les tendances épidémiologiques sont en faveur de la persistance de la maladie et un retard de diagnostic dans la prise en charge des cas de lèpre.

English abstract

## Abstract (en)

**Introduction:**

leprosy is a disease found and unevenly distributed in Chad. Since 1997, the annual national prevalence has been less than 1/10000 inhabitants, the elimination threshold set by the World Health Organization (WHO). The purpose of this study is to describe epidemiological trends of leprosy in Chad between 2015 and 2019, in order to provide the necessary data for the development of more effective strategies for leprosy control.

**Methods:**

we conducted a retrospective, descriptive study of patients with leprosy diagnosed between 2015 and 2019 at national level. Data were collected from the database of the National Program for the Control of Leprosy in Chad (NPCLC).

**Results:**

a total of 1896 new cases of leprosy were detected in Chad between 2015 and 2019. The rates of patients aged 15 to 70 years and children under 15 years were 92.08% and 7.92% respectively. Sex ratio (M/F) was 1.68. The annual average detection rate was 2.6/100 000, with an average rate of multi-bacillary leprosy of 83.10% and degree 2 disability (2DD) of 20.38%. The average rate of degree 2 disability in children under 15 years of age was 0.92% . However, our study identified five districts as endemic (Adré, Abéché, Aboudeia, Koukou, et Bebedjia) in 2019, where the prevalence rate was above 1/10000 inhabitants.

**Conclusion:**

epidemiological trends are in favor of the persistence of the disease and a delay in diagnosis and in the management of leprosy cases.

## Introduction

La lèpre est une maladie bactérienne causée par le bacille d'Hansen appartenant au complexe *Mycobacterium leprae*. Il s´agit d´une maladie infectieuse à transmission interhumaine dont la période d´incubation longue (5 à 20 ans) rend le diagnostic difficile [1]. Sur le plan clinique, la lèpre touche essentiellement la peau et les nerfs périphériques qui à long terme, peut aboutir à des déficits sensoriels et moteurs ainsi qu´à des mutilations et des déformations. Grâce à leurs conséquences esthétiques, ces lésions ont un fort impact psychologique et social pouvant induire une importante stigmatisation [1]. L'élimination de la lèpre en tant que problème de santé publique a été accomplie à l´échelle mondiale en 2000 (conformément à la résolution WHA44.9 de l´Assemblée mondiale de la Santé), au niveau du Tchad et dans la plupart des pays en 2010 [2]. L´objectif était d´obtenir une prévalence annuelle inférieure à 1 cas/10 000 habitants [3]. Les trois cibles principales à atteindre à savoir: i) l´élimination totale des nouvels cas d´incapacité de degré 2 (ID2, correspondant à la présence d´une déformation ou d´une lésion visible) chez les enfants; ii) la réduction du taux de nouveaux cas d´ID2 à moins de 1 cas pour 1 million d´habitants; iii) l´abrogation des lois permettant la discrimination des patients atteints de la lèpre dans tous les pays où ces règles étaient en vigueur [2].

Le Tchad est un pays d´Afrique Centrale avec une superficie de 1.284.000 km^2^ et une population de 16.284.000 habitants en 2019. Selon le Programme National de Lutte contre la Lèpre (PNLLT), le Tchad a atteint le seuil d´élimination de la lèpre en 1997 avec un taux de prévalence de 0,74/10000hbts [4]. On dénombre encore un nombre important de cas d´ID2 parmi lesquels des enfants de moins de 15 ans. L´objectif général de cette étude était de décrire l´épidémiologie des cas de lèpre notifiés au Tchad entre 2015 et 2019.

## Méthodes

**Type d´étude:** il s'agit d'une étude rétrospective, descriptive étalée sur une période de cinq années, entre le 1^er^ janvier 2015 et le 30 décembre 2019 ou on a recensé 1896 nouveaux cas. Le critère d´inclusion était un diagnostic de lèpre confirmé par examen clinique selon le guide de l´OMS et du PNLLT puis ont été exclus les patients en rechute, guéri et les décédés. Pour diminuer les biais, nous avons comparé les rapports annuels de PNLLT et de l´Observatoire mondial de la santé.

**Cadre de l´étude:** l´étude a été menée dans les 116 districts fonctionnels répartis dans 23 délégations provinciales sanitaires du Tchad ayant au moins un correspondant du Programme National de Lutte contre la Lèpre du Tchad (PNLLT). Le PNLLT est un organe dépendant du Ministère de la Santé ayant pour mission la prise en charge des cas de lèpre, la centralisation des données et la distribution gratuite des médicaments (Polychimiothérapie [PCT]).

**Source de données:** nous avons utilisé les bases de données du PNLLT constitué des rapports annuels et des fiches de notification des district; nous avons relevé les paramètres suivants: sociodémographique, clinique puis calculé les indicateurs épidémiologiques de la lèpre selon OMS [2].

**Variables:** les variables quantitatives ont été utilisées pour les calculs de la moyenne et les taux ensuite les variables qualitatives pour les proportions.

**Analyse des données:** les données recueillies étaient analysées grâce aux logiciels Excel 2013 et QGIS 3.16.8.

**Considérations éthiques:** la clairance éthique a été approuvée par le comité National bioéthique du Tchad sous le N°171/PR/MESRI/SG/CNBT/2019. Toutes les données ont été antonymies et la confidentialité a été strictement respectée dans le traitement et l'analyse des données.

**Source de financement:** cette étude a été entièrement financée par l'Organisation de Coordination pour la lutte contre les Endémies en Afrique Centrale (OCEAC) dans le cadre du Projet de lutte contre les maladies tropicales négligées en Afrique centrale (Projet MTN) à travers le Ministère de la Coopération Économique et du Développement (BMZ) de la République fédérale d´Allemagne par la KfW (Banque allemande de développement).

## Résultats

Sur la période d´étude (2015-2019), 1896 nouveaux cas de lèpre ont été détectés à l´échelle nationale. La moyenne d´âge des patients était de 38 ans. La proportion des patients âgés entre 15 et 70 ans et celle des enfants de moins de 15 ans était respectivement de 92,08% et 7,92%, avec un sexe ratio (H/F) de 1.68. Le taux de détection moyen annuel était de 2,6 /100 000, avec une moyenne de 83,10% de malades multibacillaires (MB) et 20.38% de patients avec une incapacité de degré 2.

**Figure 1:**
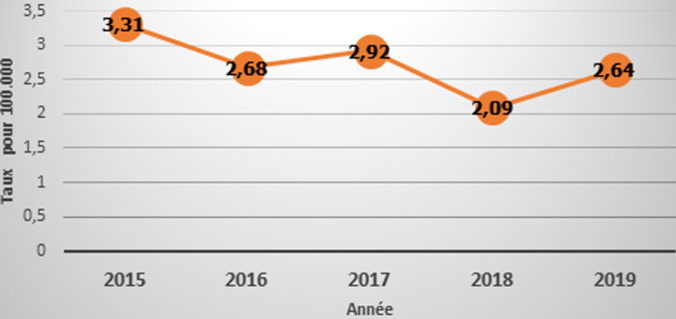
courbe évolutive de l´incidence de la lèpre au Tchad

Pour l´année 2019, le Tchad enregistrait 446 nouveau cas de lèpre, représentant un taux de prévalence de 0,26/10 000 habitants au niveau national, néanmoins 5 districts ayant un taux de prévalence supérieur à 1 cas pour 10.000/habitants. Les variations du taux de détection de la lèpre en fonction des années montraient une diminution entre 2015 et 2019 passants 3,31/100.000 habitants à 2,64/100000 habitants. Cependant, cette dynamique n´était linéaire avec une réduction progressive de l´incidence de 3,31/100.000 habitants en 2015 à 2,68/100.000 habitants en 2016, suivis d´une évolution en dent de scie entre 2016 et 2018 et d´un remonté de l´incidence en 2019 (Figure 1).

**Figure 2:**
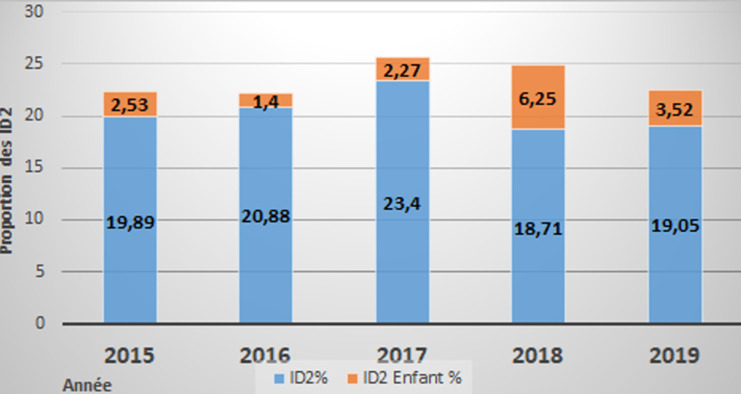
proportion de degré d´incapacité 2 (ID2%) concernant les nouveaux cas détectés chez les adultes et enfants entre 2015 et 2019 au Tchad

À l´échelle nationale, la proportion des malades présentant une incapacité de degré 2 nouvellement détectés restait relativement stable entre 2015 (19,89%) et 2019 (19,05%) avec un pic 2017. La moyenne annuelle des ID2 est de 20.38%. Chez les enfants, cette valeur d´incapacité de degré 2 montrait une augmentation importante de 2.53% à 3.25% entre 2015 à 2019 avec un point de rechute en 2016 et un pic en 2018 (Figure 2).

**Figure 3:**
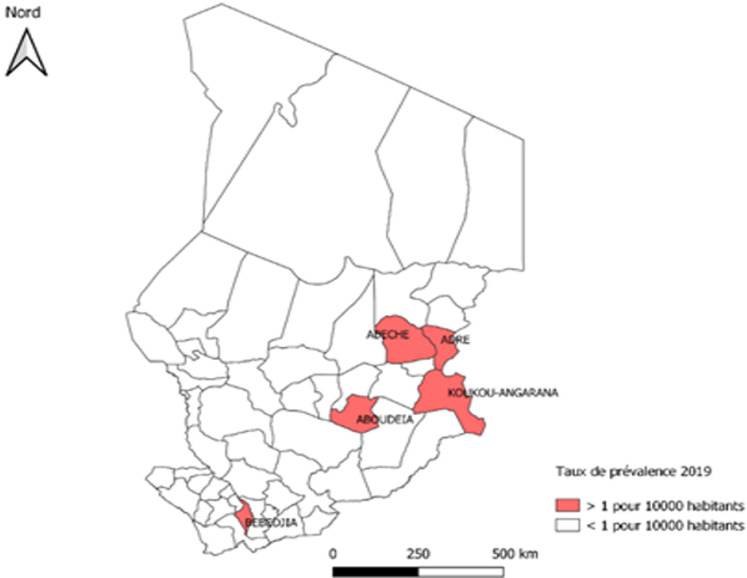
carte de prévalence de la lèpre dans les zones hyper-endémiques au Tchad par district sanitaire en 2019

Le nombre de districts hyper-endémiques est passé de 6 en 2015 à 5 en 2019 [4]. Ces 5 districts présentaient des taux de prévalence de la lèpre de 1,6/10 000 habitants à Adré,1,73/10 000 habitants à Abéché, 1,06/10 000 habitants à Aboudeia, 1,23/10 000 habitants à Koukou et 1,12/10000 habitants à Bebedjia pour l´année 2019 (Figure 3); pour les autres districts une prévalence inférieure à 1/100 000 habitants, ces taux étaient restés stagnantes pendant les 5 ans de lutte, avec peu de variation d´une année à l´autre (Figure 3).

## Discussion

Il s´agit d´une première étude rétrospective de 2015 à 2019 sur la situation de la lèpre au Tchad. Les données, considérées comme exhaustives pour les patients ayant bénéficié d´une prise en charge, ont permis de décrire l'évolution de la lutte contre la lèpre au Tchad. Le taux de prévalence de 0,26/10.000 habitants, témoigne de son élimination comme problème de santé public. Nonobstant, une sur endémicité dans cinq districts sanitaires hyper endémiques a été observée. Ceci corrobore avec les observations faites dans les études réalisées en Nouvelle-Calédonie et au Cameroun [5, 6].

Dans la présente étude, nous avons aussi noté que 92,33% des patients ont un âge compris entre 15 à 70 ans; les enfants représentant 767% de cette population de lépreux. Ces constats sont similaires à celles des études menées par Ghunawat *et al*. à Dehli [7] et Wangara *et al*. au Kenya [8], qui ont observé que les cas de lèpre infantile constituaient 7,06% et 7,5% du total des cas répertoriés. La proportion d´enfants étant considérée comme un indicateur de transmission récent de la maladie; le taux inférieur à 10% observé dans cette période d´étude correspond à une diminution de la transmission [9].

La lèpre est aussi en général observée plus fréquemment chez l´homme que chez la femme [10, 11]. Notre étude a confirmé cette tendance générale avec un sex-ratio (homme/femme) de 1.68. Cette prédominance masculine pouvant s´expliquer en partie par un accès plus facile aux hommes dans les services de santé rurale [11].

Selon les indicateurs clés de la stratégie 2016-2020 de l'OMS [2], le taux de détection a légèrement régressé de 3,31 à 2,64 pour 100.000 habitants pour le Tchad; cette tendance serait confirmée par une diminution des activités de détection et une chute de l´efficacité de la prise en charge par le PNLLT. Une augmentation de la proportion de cas chez les enfants de moins de 15 ans de 2,58 à 3.52% est une preuve de la persistance d´une transmission active de la lèpre, corroborant ainsi avec les conclusions faites dans les précédentes études menées au Bénin et au Togo [11, 12].

Les résultats obtenus ont montré que les formes MB étaient les plus fréquentes (83,10%), comparés à ceux d´une précédente étude faite au Cameroun en 2014 [6], qui indiquait que ces formes MB représentaient 87% et restaient prédominantes dans d´autres études où la lèpre a été déclarée, ne plus constituer un problème de santé publique [13]. La forte proportion des formes MB augmente les risques de contagiosité de la maladie. Ainsi que, la durée du traitement (plus longue) peut conduire à l´abandon du traitement par les patients (une augmentation des perdus de vue au cours du traitement) et par conséquent contribuer à la propagation de la maladie puis la survenue des formes résistantes.

Parmi les patients inclus, 19,05% en 2019 avaient une incapacité de degré 2 selon l´OMS. Ceci témoignant d´un diagnostic assez tardif et/ou d´une prise en charge retardée par la polychimiothérapie. Cette proportion est inférieure à celle rapportée par des études menées au Bénin qui indique un taux de 32% en 2018 [12].

La répartition géographique des cas, met en évidence le taux de prévalence supérieur à 1 cas pour 10.000 habitants dans les cinq districts sanitaires ciblés (Adré, Abéché, Aboudeia, Koukou, Bebedjia). Deux autres mécanismes qui exposent les personnes à un risque plus élevé de contracter la lèpre pourraient expliquer les taux plus élevés de transmission active continue dans les points chauds et la pauvreté [7, 10]. Il s´agit de l´affaiblissement du système immunitaire dû à l´absence d´eau potable et/ou des mesures d´hygiène appropriées, et d´une alimentation malsaine reconnue depuis longtemps comme des déterminants importants pour la survenue de la lèpre [1, 14].

L´étude que nous avons menée étant rétrospective, il devient difficile d´expliquer les variations de taux d´incidence observées entre 2015 et 2019 au Tchad, mais la tendance est presque linéaire et cela explique la faiblesse du système de prise en charge des cas de lèpre pendant cette période. Les estimations de la population ont été calculées sur la base des taux de croissance nationaux dans les districts [4]. Par conséquent, nous n´avons pas tenu compte des fluctuations, déclenchées par l´urbanisation et l´exode des populations dans les grandes villes. Le dépistage passif de la lèpre les lacunes de notification par les points focaux (faux diagnostic des perdus de vue), les difficultés d´accès des patients aux structures de soins, les pratiques de l´automédication et l´absence de la sensibilisation communautaires pourraient expliquer ses variations de l´incidence d´une année à une autre.

Les recherches futures pourraient se concentrer sur l´amélioration des stratégies de recherche de cas et du traitement de la lèpre afin optimiser davantage les stratégies d´élimination de la lèpre. Les données sur le temps de diagnostic, l´achèvement du traitement et les taux de réussite du traitement. Implication des agents de santé communautaire dans la distribution des médicaments et la sensibilisation sur les principaux signes de la lèpre pourraient être analysées, afin d´optimiser plus les programmes de lutte contre la Lèpre au Tchad.

**Limite de l´étude:** les limites de cette étude est aue l´une de faiblesses du système de surveillance sont les données manquantes sur les cas contacts, les réactions lépreuses, le retraitement et prélèvement de frottis. Ces indicateurs renseignent sur la qualité du dépistage, du traitement et du suivi des cas.

## Conclusion

La prévalence de la lèpre dans notre étude atteste l´élimination de la lèpre comme problème prioritaire de santé publique au Tchad, mais la maladie reste un défi majeur de santé dû au jeune âge de la population touchée par la maladie. La prédominance des formes multibacillaires et le nombre élevé des incapacités de degré 2, prouvent la persistance de la maladie et un retard de diagnostic dans la prise en charge des cas de lèpre au Tchad. Compte tenu de cela, il est impératif de renforcer la surveillance par la distribution des médicaments et la sensibilisation de la population par les agents de santé communautaire.

### Etat des connaissances sur le sujet

- L´élimination de la lèpre en tant que problème de santé publique a été accomplie à l´échelle mondiale en 2000 selon l´OMS;
- Des progrès ont été accomplis en matière de lutte contre la lèpre depuis l´introduction de la polychimiothérapie (PCT).

### Contribution de notre étude à la connaissance

- Une première étude au Tchad qui apporte des connaissances sur l´état de lieu de la lèpre;
- Les indicateurs étudiés sont en faveur de persistance de la lèpre malgré la lutte du PNLLT. Ceci permettra au PNLLT de changer la stratégie de lutte.

## Remerciements

Nous remercions, le Programme National de lutte contre la lèpre du Tchad, la fondation Raoul Follereau et la Mission Evangélique contre la lèpre pour la mise à disposition des données.
